# Downregulation of Plasma Membrane Ca^2+^ ATPase driven by tyrosine hydroxylase-Gal4 reduces Drosophila lifespan independently of effects in neurons

**DOI:** 10.1080/19336934.2023.2192457

**Published:** 2023-03-22

**Authors:** Brenda Erhardt, Lia Frenkel, María Silvina Marcora, María Isabel Farías, Carina Cintia Ferrari, Fernando Juan Pitossi, María Celeste Leal

**Affiliations:** a Instituto de Investigaciones Bioquímicas de Buenos Aires (IIBBA)-Consejo Nacional de Investigaciones Científicas y Técnicas (CONICET), Buenos Aires, Argentina; b Fundación Instituto Leloir, Buenos Aires, Argentina; c Laboratorio de Neurociencias del tiempo- iB3, Instituto de Biociencias, Biotecnología y Biología traslacional, Facultad de Ciencias Exactas y Naturales, Universidad de Buenos Aires, Buenos Aires, Argentina; d Departamento de Química Biológica, Instituto de Química y Fisicoquímica Biológicas (IQUIFIB)-CONICET, Buenos Aires, Argentina

**Keywords:** Calcium, gut, flies, dopaminergic neurons, GAL4-UAS, GAL80

## Abstract

In *Drosophila melanogaster*, several Gal4 drivers are used to direct gene/RNAi expression to different dopaminergic neuronal clusters. We previously developed a fly model of Parkinson’s disease, in which dopaminergic neurons had elevated cytosolic Ca^2+^ due to the expression of a Plasma Membrane Ca^2+^ ATPase (PMCA) RNAi under the thyroxine hydroxylase (TH)-Gal4 driver. Surprisingly, TH-Gal4>PMCA^RNAi^ flies died earlier compared to controls and showed swelling in the abdominal area. Flies expressing the PMCA^RNAi^ under other TH drivers also showed such swelling and shorter lifespan. Considering that TH-Gal4 is also expressed in the gut, we proposed to suppress the expression specifically in the nervous system, while maintaining the activation in the gut. Therefore, we expressed Gal80 under the direction of the panneuronal synaptobrevin (nSyb) promoter in the context of TH-Gal4. nSyb-Gal80; TH-Gal4>PMCA^RNAi^ flies showed the same reduction of survival as TH-Gal4>PMCA^RNAi^ flies, meaning that the phenotype of abdomen swelling and reduced survival could be due to the expression of the PMCA^RNAi^ in the gut. In *perimortem* stages TH-Gal4>PMCA^RNAi^ guts had alteration in the proventriculi and crops. The proventriculi appeared to lose cells and collapse on itself, and the crop increased its size several times with the appearance of cellular accumulations at its entrance. No altered expression or phenotype was observed in flies expressing PMCA^RNAi^ in the dopaminergic PAM cluster (PAM-Gal4>PMCA^RNAi^). In this work we show the importance of checking the global expression of each promoter and the relevance of the inhibition of PMCA expression in the gut.

## Introduction

In *Drosophila*, as in vertebrates, the rate-limiting step in dopamine biosynthesis is catalysed by the enzyme tyrosine hydroxylase (TH; the *pale* gene in flies). These flies present six major clusters of TH-positive neurons in each brain hemisphere: two located anteriorly (protocerebral anterior medial: PAM and protocerebral anterior lateral: PAL) and four posteriorly (protocerebral posterior lateral: PPL1 and PPL2; protocerebral posterior medial: PPM1/2 and PPM3) [1]]. There are several drivers available for activating gene expression in these dopaminergic clusters, using the Gal4-UAS [2] system. In particular, the TH-Gal4 promoter [3] drives the expression in most of these clusters (50 neurons), with the exception of the majority of the PAM group. More recently, Liu et al. created shorter promoters based on the TH-Gal4 construct that drive the expression in smaller subsets of TH+ neurons [4], which is very useful for characterizing the dopamine pathway. For instance, TH-F1-Gal4 drives expression in some neurons of the clusters PPM2, PPM3, PPL1/2 (14 neurons) while TH-D4-Gal4 does so in PPM2, PPM3 and PPL1 (11 neurons) [5]. Another driver, PAM-Gal4 (R58E02) was generated by cloning enhancer DNA sequence of positive transcription in PAM neurons [5; 6], and was used to identify circuits involving the PAM cluster, particularly the connection between this cluster and the mushroom bodies [7,8].

We previously developed a fly model to study the consequences of a sustained increment of cytosolic Ca^2+^ in the dopaminergic neurons [9] caused by the expression of a constitutive RNAi against PMCA (Plasma Membrane Calcium ATPase, PMCA^RNAi^), using the TH driver (TH-Gal4>PMCA^RNAi^). PMCA is a transmembrane protein that pumps Ca^2+^ from cytosol to the extracellular media. In the absence of this pump, dopaminergic neurons showed elevated levels of intracellular Ca^2+^. This condition prompted an increase in ROS together with alterations in dopamine levels and motor behaviour.

Even though TH-Gal4 is widely used as a specific promoter for dopaminergic neurons, it also promotes expression in some regions of the gut [3]. Interestingly, the *pale* gene, from which the TH-Gal4 construct originated, is expressed in the nervous system and hypoderm [10]. It is relevant when devising a model for nervous system pathology and using several drivers to direct the expression to the brain, to take into account the expression in different parts of the animals, such as the gut. To the best of our knowledge, there are no published detailed reports on TH-Gal4 expression in adult flies.

Here, we describe gut alterations in our TH-Gal4>PMCA^RNAi^ model. These flies showed a swollen abdomen and died earlier compared to controls. This data may be useful to consider when developing new fly models for neurological diseases.

## Materials and methods

### *Drosophila* strains and maintenance

Fly stocks and crosses were raised on standard cornmeal-yeast-agar medium under a 12 h/12 h light/dark cycle in 4-inch plastic vials. Flies were transferred to a fresh food medium every 2–4 days. Unless otherwise stated, crosses were done at 25°C and experiments carried out using 8- to 10-day-old males maintained at 28°C (20 males/vial). w1118 #5905, TH (pale)-Gal4 #8848, UAS mCD8:mCherry #27391 and UAS EGFP #6874 were obtained from Bloomington Stock Center; UAS PMCA^RNAi^ #101743 was obtained from Vienna *Drosophila* RNAi Center; TH-F1-Gal4 and TH-D4-Gal4 were gently shared by Wu [5], and PAM-Gal4 was gently shared by Tanimoto [5].

## Survival assay

Adults were collected within 24 h after hatching of the imago and a total of 84–268 males per experimental group were monitored for survival, evaluating the number of dead flies every 2 days. This data is presented in a curve showing the percentage of flies alive and the average lifespan of each genotype using the Kaplan-Meier approach, according to [11] with some modifications.

## Swelling phenotype and gut dissection

Flies were anesthetized with CO_2_ and pictures of the entire body of the flies were taken with an Olympus MVX10 stereo microscope. In order to study the expression pattern of the TH-Gal4 driver and to measure the size of the crop, flies were anesthetized with CO_2_ and their organs were dissected in phosphate buffered saline (PBS) and mounted in Mowiol. Digital images were obtained with a 4× (NA 0.13) objective in an Olympus B×60 microscope and crops’ areas were quantified using FIJI [12]. The same dissection procedure was used for the comparative expression pattern analysis of TH-Gal4, TH-F1-Gal4, TH-D4-Gal4 and PAM-Gal4. In this case, the digital images were obtained using a 20× (NA 0.8) objective, except for the images of the hindgut, which were obtained with a 10× (NA 0.45) objective in a Zeiss LSM 880 confocal microscope. For proventriculi morphology analysis of PMCA^RNAi^-expressing flies, the same dissection and mounting procedure was used, but the images were obtained with a 5× objective (NA 0.15) in a Zeiss Observer 3 microscope. To further study the proventriculi at higher magnifications, after dissection, proventriculi were fixed for 20 min in 4% formaldehyde in phosphate buffer (PB) at room temperature. Cell nuclei were stained with Hoechst for 10 min, proventriculi were mounted in Mowiol and images were taken with a 40× water immersion objective (NA 1.2) using Zeiss LSM 880 confocal microscope. Finally, the expression pattern of Gal4 was studied in flies in which its expression was specifically suppressed in neurons using nSyb-Gal80. After being anesthetized with CO_2_, these flies’ whole bodies were fixed in 4% formaldehyde in PB for 1.5 h at room temperature. The organs were then dissected in PB-Tx 0.3% and mounted in Mowiol. Images were taken with a 20× objective (NA 0.8) using Zeiss LSM 880 confocal microscope.

## Statistical analysis

Analyses were performed using GraphPad Prism version 8.0.0 for Windows, (GraphPad Software, San Diego, California, USA). Results are presented as mean ± SEM. Two-way ANOVA was performed with Tukey’s multiple comparisons test. The tests used in each figure are detailed in figure legends. Statistical significance level was α = 0.05.

## Results

### The expression of PMCA^RNAi^ under the TH-Gal4, but not PAM-Gal4 driver decreased fly lifespan

**Figure 1.**
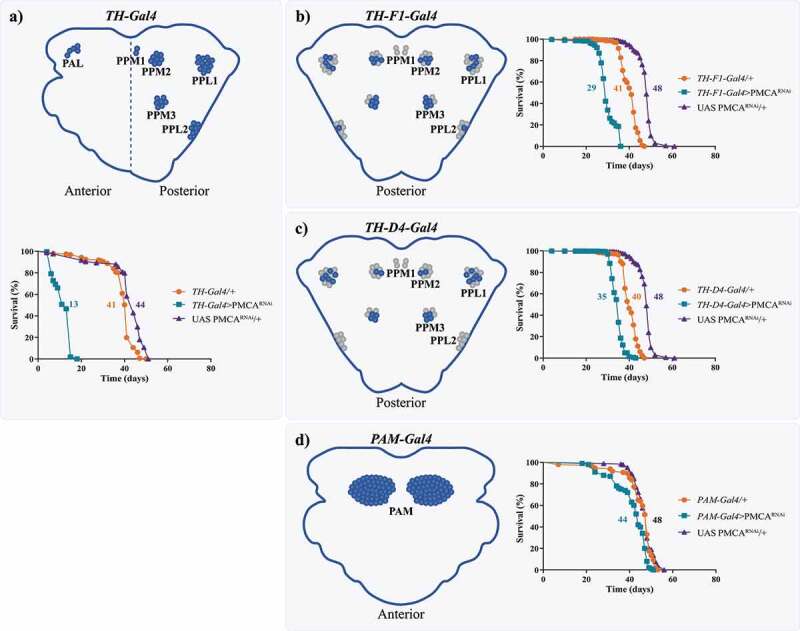
The expression of PMCA^RNAi^ under dopaminergic drivers, with the exception of PAM-Gal4 decreased flies’ lifespan. Diagrams show the brain dopaminergic neurons in which each promoter is expressed. a, TH-Gal4 is active in several dopaminergic clusters in the anterior and posterior regions of the brain. The expression of TH-Gal4>PMCA^RNAi^ markedly reduced lifespan compared to controls. b and c, TH-F1-Gal4 and TH-D4-Gal4 are active in 28 and 22 neurons, respectively, and, when used to drive expression of PMCA^RNAi^, also presented a lower survival rate, but higher than TH-Gal4>PMCA^RNAi^ flies. d, Flies expressing PMCA^RNAi^ under the PAM-Gal4 promoter showed only minor differences in lifespan compared to its controls (with a mean lifespan similar to most other controls). All curves were analysed with the Log-rank Mantel Cox test and each curve was replicated 2 or 3 times.

The expression of PMCA^RNAi^ driven by TH-Gal4 induced motor alterations even in the absence of evident neurodegeneration [9]. Thus, we decided to characterize dopaminergic status over time, in order to evaluate the presence of more subtle neuronal alterations. Surprisingly, we found that TH-Gal4>PMCA^RNAi^ flies died earlier, compared to controls (mean lifespan: TH-Gal4>PMCA^RNAi^ 13 days; TH-Gal4/+ 41 days; UAS PMCA^RNAi^/+ 44 days) (Figure 1a). We evaluated whether the expression of PMCA^RNAi^ under other dopaminergic neuron drivers also decreased survival (Figure 1b-d). Both TH-F1-Gal4>PMCA^RNAi^ and TH-D4-Gal4>PMCA^RNAi^ flies showed shorter lifespan compared to their respective controls (TH-F1-Gal4>PMCA^RNAi^ 29 days; TH-F1-Gal4/+ 41 days; UAS PMCA^RNAi^/+ 48 days; Figure 1b) (TH-D4-Gal4>PMCA^RNAi^ 35 days; TH-D4-Gal4/+ 40 days; UAS PMCA^RNAi^/+ 48 days; Figure 1c). On the other hand, PMCA^RNAi^ expressed in the PAM cluster showed the smallest difference with its controls (PAM-Gal4>PMCA^RNAi^ 44 days; PAM-Gal4/+ 48 days; UAS PMCA^RNAi^/+ 48 days). Consequently, we suspected that this could be produced by a differential expression of Gal4 in the gut between the TH and PAM drivers.

## The TH-Gal4 driver activated reporter expression in the proventriculus, crop and hindgut

**Figure 2.**
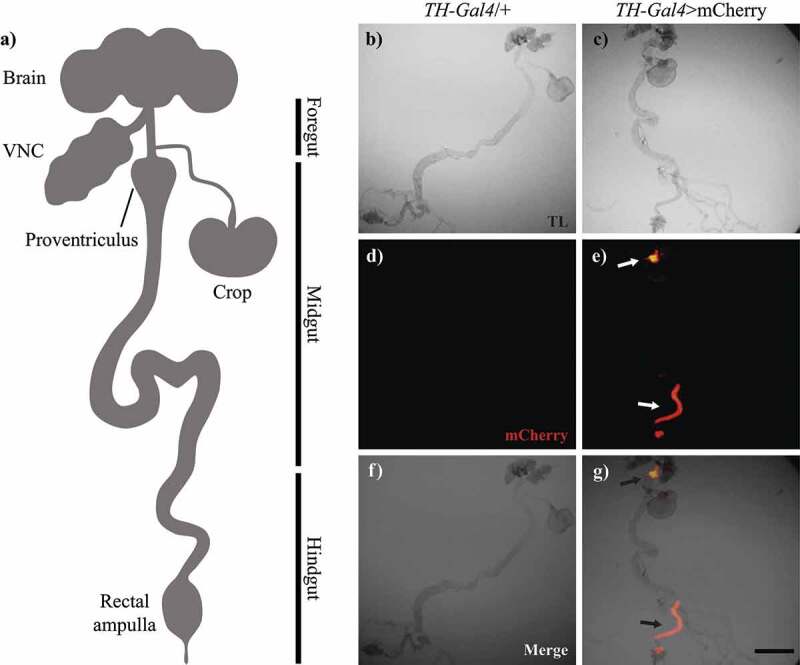
TH-Gal4 was expressed in the digestive tract. a, Schematic representation of the central nervous system and digestive tract of *Drosophila melanogaster* (VNC: ventral nerve cord). Microscopy images of the gut from TH-Gal4/+ and TH-Gal4>mcherry flies: b-c, Bright field; d-e, mCherry fluorescence; and f-g, merge. Red fluorescence was detected only in the proventriculus and hindgut (arrows, e and g) from TH-Gal4>mcherry flies. Scale bar: 250 µm.

We analysed the expression pattern of mCherry driven by TH-Gal4 in the adult gut of flies (Figure 2). Red fluorescence was detected in the proventriculus, crop entrance and hindgut of TH-Gal4>mCherry flies, but not in TH-Gal4/+ (control) flies (Figure 2d-g). Under transmitted light, we found no obvious differences in gut morphology between groups (Figure 2b-c).

## TH-F1-Gal4 and TH-D4-Gal4 drivers activated expression in both the proventriculus and hindgut, whereas PAM-Gal4 expression was not detected in the gut

**Figure 3.**
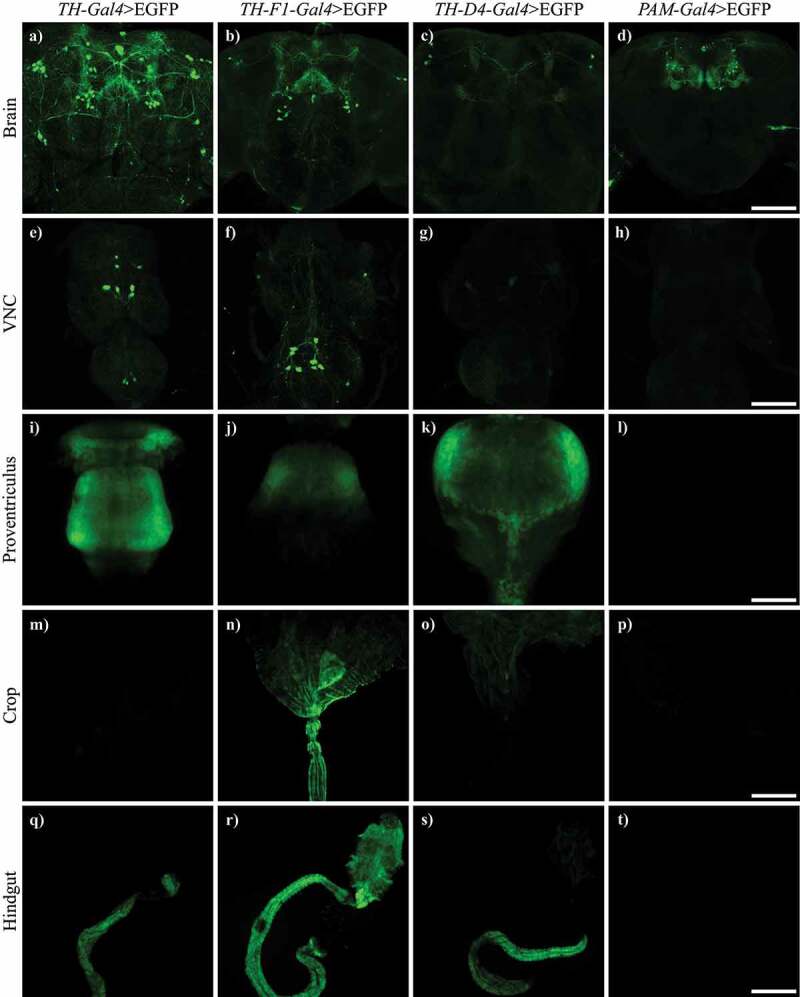
TH-F1-Gal4 and TH-D4-Gal4, but not PAM-Gal4, also showed expression in the gut. Confocal images showing expression of EGFP driven by TH-Gal4, TH-F1-Gal4, TH-D4-Gal4 and PAM-Gal4 in brain (a-d), VNC (e-h), proventriculus (i-l), crop (m-p) and hindgut (q-t). VNC: central nervous cord. Scale bar: d, h and p, 100 µm; l, 50 µm and t, 200 µm.

Since fluorescence in the gut of TH-Gal4>mCherry flies showed expression of TH-Gal4 in that tissue, we decided to study the expression patterns of other drivers used for targeting dopaminergic neurons. We analysed and compared the expression of EGFP under TH-Gal4, TH-F1-Gal4, TH-D4-Gal4 and PAM-Gal4 drivers in the brain, ventral nerve cord (VNC) and gut. We found that TH-F1-Gal4 and TH-D4-Gal4 drivers were activated in the dopaminergic neurons, as reported, and VNC, but also in the proventriculus and the hindgut, in a manner similar to TH-Gal4. On the contrary, fluorescence in PAM-Gal4>EGFP flies was limited to the PAM neuronal cluster (Figure 3 and Table 1).

**Table 1.** Qualitative summary of the activation pattern of TH-Gal4, TH-F1-GAL4, TH-D4-GAL4, PAM-GAL4 and TH-Gal4/nsyb-Gal80 and of *perimortem* bloating and survival when PMCA^RNAi^ is expressed.

	TH-Gal4	TH-F1-Gal4	TH-D4-Gal4	PAM-Gal4	TH-Gal4/nSyb-Gal80
VNC	++	++	-	-	-
Proventriculus	+++	++	+++	-	+++
Crop	+	+++	+	-	+
Hindgut	++	+++	++	-	++
*Perimortem* bloating when PMCA^RNAi^ is expressed	++	+	+	-	+
Reduction in mean lifespan when PMCA^RNAi^ is expressed	13 vs. 41 TH-Gal4/+;44 UAS-PMCA^RNAi^	29 vs. 41 TH-F1-Gal4/+; 48 UAS-PMCA^RNAi^	35 vs. 40 TH-F1-Gal4/+; 48 UAS-PMCA^RNAi^	44 vs. 48 PAM-Gal4/+; 48 UAS-PMCA^RNAi^	8 vs. 39 nSybGal80,TH-Gal4/+; 44 TH-Gal4/+; 44 UAS PMCA^RNAi^;nSybGal80

## TH-Gal4>PMCA^RNAi^ flies showed a *perimortem* abdominal bloating phenotype that did not reverse when PMCA^RNAi^ expression was silenced in the nervous system

**Figure 4.**
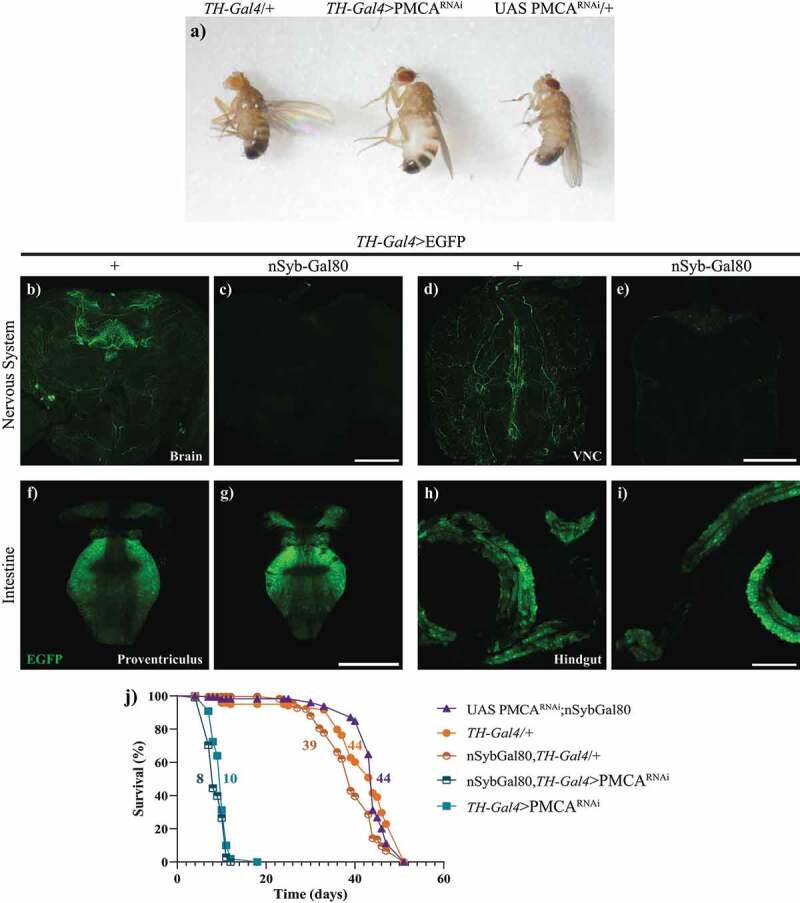
TH-Gal4>PMCA^RNAi^ flies showed a *perimortem* swelling of the abdomen and suppressing neuronal expression of PMCA^RNAi^ did not prevent the shortening of their lifespan. a, Abdominal swelling phenotype in 10 day-old TH-Gal4>PMCA^RNAi^ flies, compared to TH-Gal4/+ and UAS PMCA^RNAi^/+ controls. b-i, Confocal images showing that nSyb-Gal80 suppresses TH-Gal4 driven EGFP expression in the brain (b,c) and the VNC (d,e), but not in the proventriculus (f,g) and hindgut (h,i). j, the lifespan curve of nSyb-Gal80;TH-Gal4>PMCA^RNAi^ flies was similar to that of TH-Gal4>PMCA^RNAi^ flies. Scale bars: 100 µm.

The analysis of TH-Gal4>PMCA^RNAi^ adult flies showed individuals with swollen abdomens (Figure 4a), compared to controls, accompanied by an exponential decrease in overall movement in *perimortem* stages, from approximately one day to hours before death. To evaluate whether this bloating abdomen phenotype and the shorter lifespan of these flies were caused by the expression of TH-Gal4>PMCA^RNAi^, either within or outside the nervous system, we silenced PMCA^RNAi^ expression only in the nervous system. To achieve this, we expressed Gal80 under a pan-neuronal synaptobrevin promoter (nSyb-Gal80) in TH-Gal4 flies (nSyb-Gal80;TH-Gal4). First, we verified that nSyb-Gal80 suppressed Gal4 activity only in the nervous system and not in the periphery, expressing EGFP in this context (nSyb-Gal80;TH-Gal4>EGFP). These flies showed a suppression of the EGFP fluorescence in the brain and VNC, compared to TH-Gal4>EFGP controls (Figure 4b-e), but still showed fluorescent signals in the proventriculus and hindgut (Figure 4f-i). This indicates that the nSyb-Gal80 was effective in suppressing Gal4 activation only in the nervous system. Knowing this, we analysed the survival of the nSyb-Gal80;TH-Gal4>PMCA^RNAi^ flies. These flies showed the same reduced lifespan and *perimortem* abdominal bloating as TH-Gal4>PMCA^RNA^ flies, suggesting that these phenotypes could be due to PMCA^RNAi^ expression in non-neuronal cells, probably in the gut (Figure 4j and Table 1).

## TH-Gal4>PMCA^RNAi^ flies showed a *perimortem* crop enlargement and shrinking of the proventriculus

**Figure 5.**
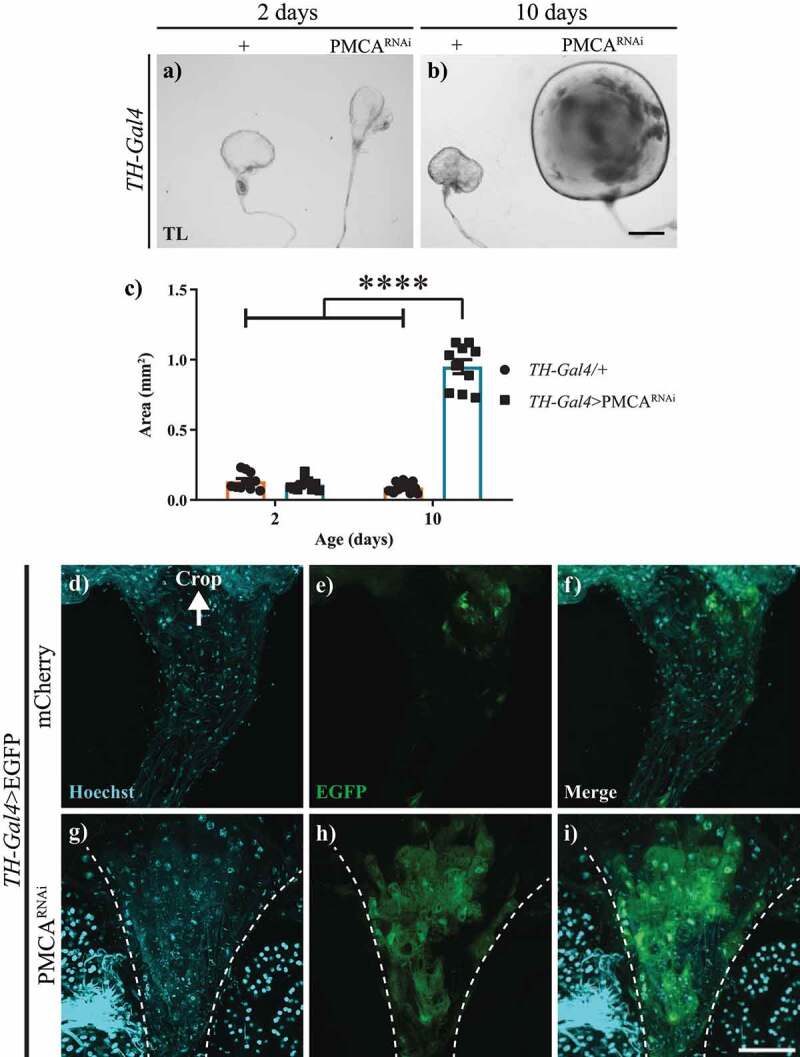
10-days-old, but not 2-days-old, TH-Gal4>PMCA^RNAi^ flies presented an increase in crop size and abnormal accumulation of cells at the entrance of the crop. a-b, Bright field microscopy images of the crops from 2 (a) and 10-days-old (b) TH-Gal4/+ (control) and TH-Gal4>PMCA^RNAi^ flies. c, Quantification of crop sizes (mm^2^) showed no difference at 2 days of age, whereas at 10 days, those from TH-Gal4>PMCA^RNAi^ flies were about 10 times larger than those from TH-Gal4/+ controls. d-i, Confocal microscopy images of the crop entrance (dotted lines) from TH-Gal4>egfp/mCherry (d-f) and TH-Gal4>EGFP/PMCA^RNAi^ (g-i). The latter showed fragmented nuclei (g) and increased EGFP fluorescence (h, merge in i) compared to the former (control). Nuclei outside the dotted line belong to a different section of the intestine that was left next to the crop in the dissection. Two-Way ANOVA Tukey’s multiple comparisons test; 10-days-old TH-Gal4>PMCA^RNAi^ versus 10-days-old TH-Gal4/+, 2-days-old TH-Gal4/+ and 2-days-old TH-Gal4>PMCA^RNAi^, ****p > 0,0001. Scale bar: 300 µm.

**Figure 6.**
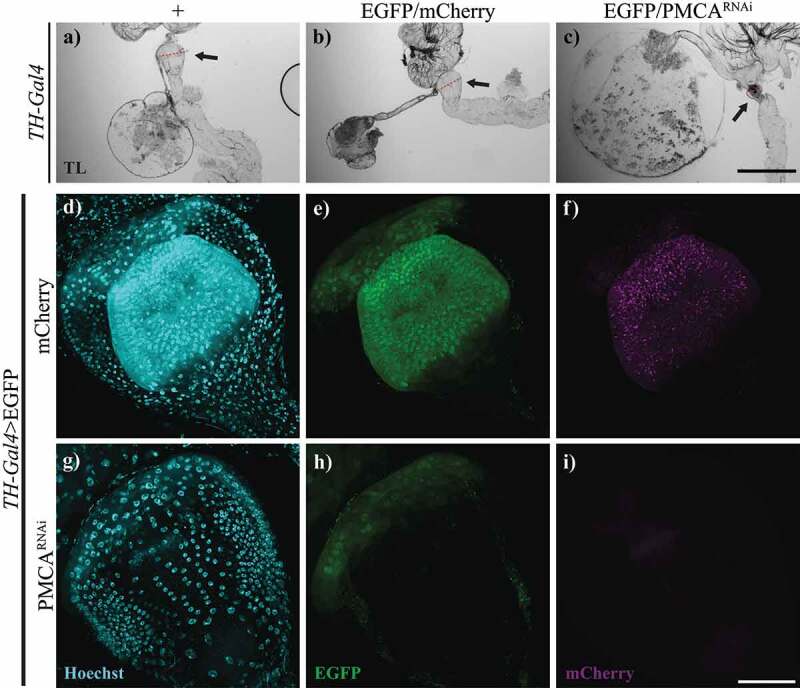
10-days-old TH-Gal4>PMCA^RNAi^ flies showed shrinking and cell loss in the proventriculus. a-c, Bright field microscopy images of proventriculi (arrows) from TH-Gal4/+ (a), TH-Gal4>egfp/mCherry (b) and TH-Gal4>EGFP/PMCA^RNAi^ (c) 10-days-old flies. The latter exhibited *perimortem* collapsing of the proventriculus, which was not evident in the controls. The diameter (indicated with a red line) of the proventriculus shown were 218,48 µm (a), 220.82 µm (b) and 131.93 µm (c). d-i, Confocal images of proventriculi from TH-Gal4>egfp/mCherry (d-f) and TH-Gal4>EGFP/PMCA^RNAi^ (g-i) flies, which presented a decreased number of nuclei and EGFP fluorescence. f and i show mCherry fluorescence, represented in magenta (no fluorescence shown in TH-Gal4>EGFP/PMCA^RNAi^ flies as they do not express mCherry). Scale bar: c, 500 µm; i, 50 µm.

A more detailed observation of the guts of TH-Gal4>PMCA^RNAi^ adult flies revealed alterations in crops and proventriculi (Figures 5 and 6), but no alterations in the hindgut (data not shown). At 10 days of age, TH-Gal4>PMCA^RNAi^ flies presented enlarged crops, 20 times the size of those from control flies of the same age (Figure 5a-c). This difference was not present in 2-days old flies. In depth analyses of the entrance of the crop in TH-Gal4>EGFP/PMCA^RNAi^ flies showed that they had an apparent abnormal accumulation of cells with a disorganization of the nuclei accompanied by an increased EGFP signal (Figure 5g-i). On the other hand, the initial sections of the crop of TH-Gal4>EGFP/mCherry controls showed only a weak expression of EGFP (Figure 5d-f). In these experiments, mCherry was used to balance the amount of UAS-construct between genotypes.

As the proventriculus’ function is to control food entry from the crop, we also studied the appearance and size of the proventriculi from TH-Gal4>EGFP/PMCA^RNAi^ flies under transmitted light. These proventriculi were smaller and appeared condensed (Figure 6c) compared to controls (TH-Gal4/+ and TH-Gal4>EGFP/mCherry, Figure 6a-b). To study the *perimortem* proventriculi structure, we expressed EGFP under TH-Gal4 and examined flies’ guts in a stage before the onset of the bloating, at 8–10 days old. Proventriculi from TH-Gal4>EGFP/PMCA^RNAi^ flies showed a striking decrease in EGFP fluorescence and number of cells (Figure 6g-i) compared to TH-Gal4>EGFP/mCherry controls (Figure 6d-f).

## Discussion

Here, we described a swollen abdomen phenotype in adult flies when PMCA^RNAi^ is expressed under the TH-Gal4 driver. This driver and its derivatives have been used in several reports to study mature dopaminergic neurons [1,13,14], however, their expression in the periphery remains largely unexplored. The initial description of TH-Gal4 by [3]using whole-mount *in situ* hybridization with an antisense Gal4 RNA in fly embryos showed labelled segments that would later form the gut in the adult. In Table 1 we summarize the relative expression levels of several drivers for dopaminergic neurons in VNC, proventriculus, crop and hindgut, and whether PMCA^RNAi^ expression in these tissues produced a concomitant phenotype. Flies expressing PMCA^RNAi^ under all TH-derived drivers presented *perimortem* bloating in the abdomen and shorter lifespan. Interestingly, TH-Gal4>PMCA^RNAi^ generated the most acute phenotype, compared with the other two shorter TH drivers. The expression of Gal80 (which suppresses Gal4 activity) in the nervous system did not revert the swollen abdomen and shorter lifespan phenotypes, suggesting that these alterations could be due to PMCA^RNAi^ expression in non-nervous cells related to the gut. The TH-Gal4 driver contains the whole *Drosophila* TH gene (with its complete regulatory region, including introns), with Gal4 and a 3’ translation termination element inserted in the middle of the first exon [3]. On the other hand, TH-F1-Gal4 and TH-D4-Gal4 are also derived from the TH gene but contain shorter regions of the TH genomic locus [5]. Flies expressing PMCA^RNAi^ under PAM-Gal4 showed subtle or inexistent alterations in the gut and mean lifespan, probably because this driver contains a regulatory sequence derived from the first intron of the *Drosophila* dopamine transporter gene [5] and was not active in the gut. These variations in the driver constructs could explain the differential expression in the periphery, and their associated phenotypes, as well as TH-Gal4 and PAM-Gal4 activation in distinct dopaminergic neuron clusters.

The conspicuous swollen abdomen phenotype of TH-Gal4>PMCA^RNAi^ flies was caused by the enlargement of the crop. We speculate it could be the consequence of an accumulation of food in the crop, prompted by a reduction of the function of proventriculus as a valve. Another possibility is that crop enlargement was rather due to increased proliferation of gastric stem cells in the proventriculus and cell migration to the crop, as a result of sustained high levels of Ca^2+^ [15], which would explain the elevated number of nuclei we observed in the crop entrance of these flies. Our results in Figure 5 g-i favours this last possibility. Our work does not provide direct evidence that silencing of PMCA in the intestine causes an increase in Ca^2+^, however, Deng et al. demonstrated that Ca^2+^ levels are elevated in the in the midgut due to silencing of PMCA by an RNAi [16]. Thus, an increase of Ca^2+^ in the gut could be a possible component of the mechanism of the PMCA action shown in our work.

The present study falls short to determine which structure/s of the gut is/are responsible for the observed phenotypes. Further experiments focused on the gut are needed to deepen the knowledge of the consequences of the downregulation of PMCA in this tissue. A screen for gut-specific drivers with a similar peripheral expression pattern to TH-Gal4 (without neuronal activation), would be useful to fully separate CNS and gut-driven phenotypes. Additionally, finding organ-specific drivers could help determine which structure/s of the gut is/are responsible for the phenotypes we described. Likewise, Ca^2+^ measurements using organ-specific drivers would contribute to understanding the influence of Ca^2+^ increments on each organ, as the possible relevance of gut Ca^2+^ levels elicited by PMCA downregulation for the phenotypes described is not totally elucidated by this work.

In summary, with these results we contribute to the understanding of the expression pattern of 4 dopaminergic neuronal drivers in adult flies: TH-Gal4, TH-F1-Gal4, TH-D4-Gal4 and PAM-Gal4. Weaver et al. showed that many drivers that are active in the nervous system are also active in the gut and vice versa [17]. It is important to be aware of the whole expression of the driver selected to answer an experimental question and the possible out-of-target consequences of using that driver. Additionally, we also described the phenotypes generated by the downregulation of PMCA in non-neuronal cells and we showed that the phenotypes in the gut were independent from PMCA^RNAi^ expression in the nervous system.

## Data Availability

The data generated during the current study are available from the corresponding author on request.
